# Advances in basic research on post-cardiac arrest syndrome in adults: a comprehensive review

**DOI:** 10.3389/fmed.2026.1770983

**Published:** 2026-03-06

**Authors:** Rui Liu, Junlong Wang

**Affiliations:** 1 Department of Emergency Medicine, The Second Affiliated Hospital of Zhejiang Chinese Medical University, Hangzhou, China; 2 Department of Urology, The First Affiliated Hospital of Zhejiang Chinese Medical University (Zhejiang Provincial Hospital of Chinese Medicine), Hangzhou, China

**Keywords:** biomarkers, ischemia-reperfusion injury, mitochondrial dysfunction, oxidative stress, post-cardiac arrest syndrome, systemic inflammatory response

## Abstract

Post-cardiac arrest syndrome (PCAS) represents a critical systemic ischemia-reperfusion injury occurring after the return of spontaneous circulation in patients who have experienced cardiac arrest. This syndrome encompasses multiple organ dysfunctions and involves complex pathophysiological mechanisms that remain incompletely understood. Despite advances in clinical management, high morbidity and mortality rates persist, underscoring the urgent need for deeper mechanistic insights and novel therapeutic strategies. Current basic research has increasingly focused on elucidating the cellular and molecular alterations underpinning PCAS, including oxidative stress, mitochondrial dysfunction, and systemic inflammatory responses. Animal models have been instrumental in mimicking the human condition, enabling the exploration of potential biomarkers and therapeutic targets. This review systematically summarizes recent progress in the fundamental research of PCAS, highlighting key findings related to its pathophysiology, molecular signaling pathways, and experimental interventions. By integrating these insights, this article aims to provide a comprehensive theoretical foundation to guide future translational research and improve clinical outcomes in PCAS management.

## 1 Introduction

Post-cardiac arrest syndrome (PCAS) represents a significant challenge in the management of patients who have experienced cardiac arrest. It is characterized by a complex interplay of physiological responses that can lead to multi-organ dysfunction and significantly impact survival and neurological outcomes. The pathophysiology of PCAS is multifaceted, involving global brain injury, myocardial dysfunction, and systemic inflammatory responses that occur as a consequence of ischemia and subsequent reperfusion following cardiac arrest. This intricate syndrome not only affects the immediate recovery of patients but also influences long-term prognosis, making it a crucial focus for both research and clinical practice (1).

Advancements in resuscitation techniques have underscored the need for a deeper understanding of the mechanisms underlying PCAS. As resuscitation strategies improve, so too does the potential for patients to survive the initial event, yet many of these survivors experience profound neurological deficits and other complications associated with PCAS. The complexity of the syndrome necessitates a multidisciplinary approach to management, incorporating strategies that address the diverse aspects of this condition, including hemodynamic stabilization, temperature management, and the prevention of secondary injuries (2).

**Figure 1:**
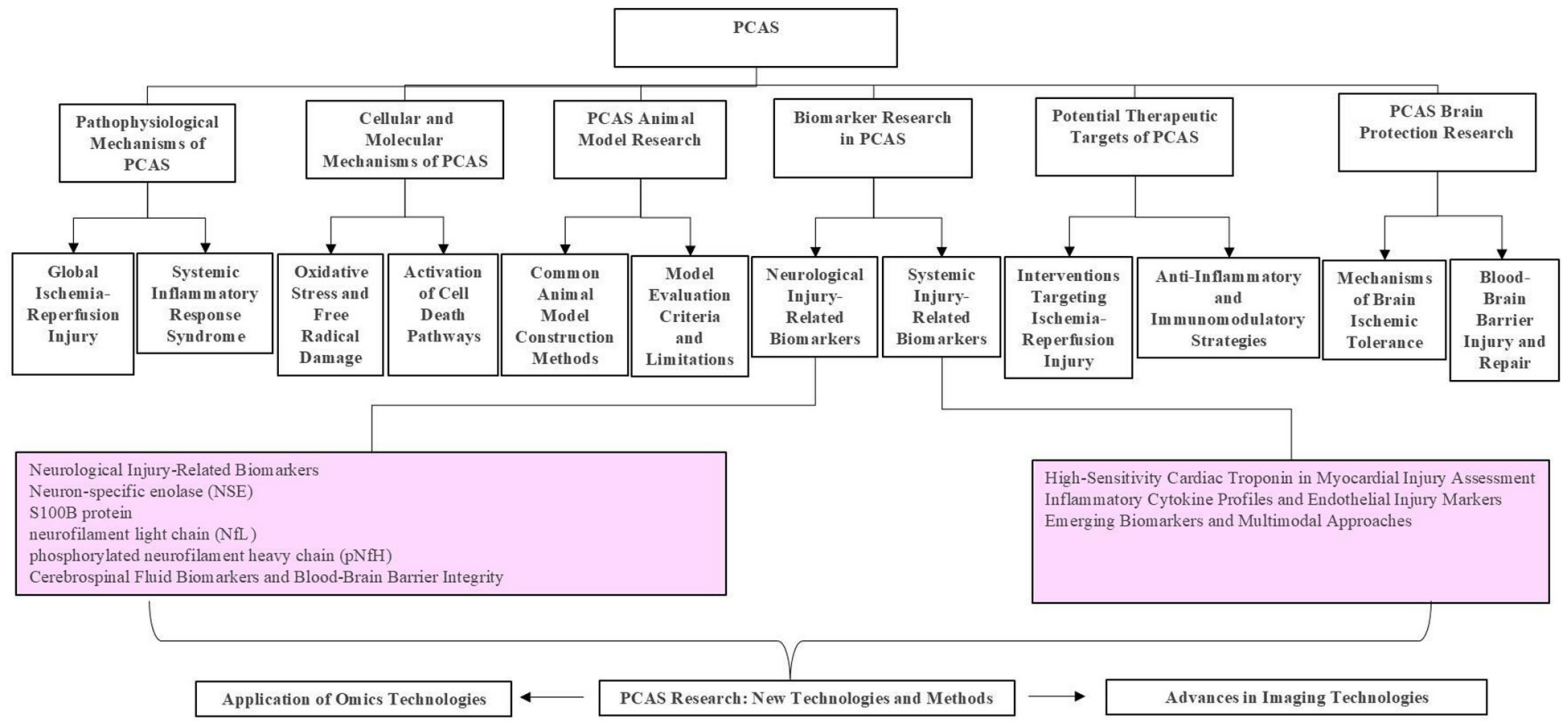
Advances in basic research on PCAS-from mechanistic studies and animal models to multi-omics analyses and targeted therapies.

Recent research has highlighted the role of molecular biology and cellular signaling pathways in elucidating the mechanisms of PCAS. For instance, studies have indicated that systemic inflammation and endothelial dysfunction are critical components that contribute to the severity of organ injury post-cardiac arrest. These findings have opened new avenues for potential therapeutic interventions aimed at modulating the inflammatory response and enhancing vascular function, which may ultimately improve patient outcomes (3). The development of targeted therapies that focus on these pathways is an area of active investigation, with the hope that they can mitigate the deleterious effects of ischemia-reperfusion injury on vital organs (Figure 1).

Furthermore, understanding the molecular underpinnings of PCAS has led to the identification of various biomarkers that may serve as prognostic indicators. For example, elevated levels of inflammatory cytokines have been associated with worse outcomes in patients following cardiac arrest, suggesting that monitoring these biomarkers could provide valuable insights into the patient's condition and guide therapeutic decisions (4). The integration of biomarker analysis into clinical practice could enhance the ability to predict outcomes and tailor interventions more effectively.

This article aims to provide a comprehensive overview of the current state of basic research on PCAS, focusing on its molecular mechanisms and potential therapeutic strategies. By synthesizing recent findings from the literature, we hope to shed light on the evolving understanding of this complex syndrome and highlight areas where further research is needed to improve management and outcomes for patients who survive cardiac arrest. The exploration of novel therapeutic approaches, including the use of inhaled gases and immunomodulatory agents, represents an exciting frontier in the quest to enhance recovery and minimize the long-term consequences of PCAS (1, 5).

Given the rapidly expanding literature on post-cardiac arrest syndrome (PCAS), this review does not aim to provide an exhaustive catalog of all reported mechanisms. Instead, we deliberately focus on key pathophysiological processes with the greatest translational relevance, particularly systemic ischemia–reperfusion injury, inflammation-driven multi-organ dysfunction, and the persistent gap between robust preclinical discoveries and limited clinical translation. By emphasizing these aspects, we aim to provide a more focused and clinically meaningful synthesis rather than a comprehensive descriptive overview.

### 1.1 Methods: literature search and study selection

This review was conducted in accordance with established recommendations for narrative reviews, including the Scale for the Assessment of Narrative Review Articles (SANRA), and with reference to the Preferred Reporting Items for Systematic Reviews and Meta-Analyses (PRISMA) 2020 statement where applicable.

A comprehensive literature search was performed in PubMed, Web of Science, and Scopus databases from inception to December 2025. The search strategy combined Medical Subject Headings (MeSH) and free-text terms related to post-cardiac arrest syndrome and its underlying mechanisms, including “post-cardiac arrest syndrome,” “cardiac arrest,” “ischemia–reperfusion injury,” “oxidative stress,” “inflammation,” “mitochondrial dysfunction,” “biomarkers,” and “therapeutic interventions.”

Original experimental studies, observational clinical studies, and interventional trials focusing on adult populations were considered eligible. Reviews, case reports, conference abstracts, and non-English publications without available translations were excluded. Studies were screened based on title and abstract, followed by full-text assessment for relevance. Study selection was performed independently by both authors, with discrepancies resolved through discussion and consensus.

Given the heterogeneity of study designs and outcomes, a qualitative synthesis approach was adopted rather than formal meta-analysis. Particular emphasis was placed on studies with clear mechanistic insights, translational relevance, and potential clinical implications for the management of post-cardiac arrest syndrome. Generative artificial intelligence tools were not used for literature selection, data extraction, or scientific content generation in this review. AI-assisted tools were used only for preliminary visual structuring of schematic illustrations under direct author supervision.

## 2 Pathophysiological mechanisms of post-cardiac arrest syndrome (PCAS)

### 2.1 Global ischemia-reperfusion injury

Cardiac arrest results in an abrupt cessation of systemic perfusion, leading to whole-body ischemia. The subsequent restoration of spontaneous circulation (ROSC) initiates reperfusion, which paradoxically exacerbates tissue injury through a complex cascade collectively referred to as global ischemia–reperfusion injury (IRI). During the ischemic phase, oxygen deprivation disrupts mitochondrial oxidative phosphorylation, leading to ATP depletion, ionic imbalance, and accumulation of metabolic by-products. Reperfusion, while essential for tissue survival, triggers a burst of reactive oxygen species (ROS) production that amplifies cellular injury.

Excessive ROS generation during reperfusion induces widespread cellular damage, simultaneously impairing mitochondrial function, disrupting macromolecular integrity, and amplifying inflammatory signaling cascades. Endothelial cells within the microcirculation are particularly vulnerable to IRI, resulting in endothelial dysfunction characterized by increased vascular permeability, impaired vasomotor regulation, and loss of barrier integrity. These alterations contribute to microcirculatory failure, including the “no-reflow” phenomenon, in which capillary perfusion remains inadequate despite restoration of macroscopic blood flow.

Microvascular obstruction is further exacerbated by leukocyte adhesion, platelet aggregation, and activation of the coagulation cascade, collectively promoting tissue hypoxia and organ dysfunction. Experimental models of cardiac arrest consistently demonstrate that microcirculatory impairment is a key determinant of post-cardiac arrest brain injury, myocardial dysfunction, and multiple organ failure. However, despite robust mechanistic evidence from preclinical studies, therapeutic strategies targeting global IRI—such as antioxidant administration or microcirculatory modulation—have not yet translated into consistent clinical benefit, underscoring a critical gap between experimental insights and bedside application (6–8).

### 2.2 Systemic inflammatory response syndrome

Following global ischemia–reperfusion injury, cardiac arrest triggers a profound systemic inflammatory response that represents a central component of post-cardiac arrest syndrome (PCAS). Tissue ischemia and reperfusion lead to the release of damage-associated molecular patterns (DAMPs), which activate pattern recognition receptors, including Toll-like receptors, on innate immune cells. This activation initiates a coordinated inflammatory cascade characterized by the rapid release of pro-inflammatory cytokines such as tumor necrosis factor-α, interleukin (IL)-1β, IL-6, and IL-8.

Concurrently, activation of the complement system further amplifies systemic inflammation through generation of anaphylatoxins and membrane attack complexes, promoting leukocyte recruitment, endothelial activation, and microvascular injury. The close interplay between inflammation and coagulation establishes a self-perpetuating cycle in which inflammatory mediators enhance coagulation activation, while coagulation pathways reciprocally intensify inflammatory signaling. This process contributes to microvascular thrombosis, impaired tissue perfusion, and progressive organ dysfunction.

At the cellular level, activated neutrophils and monocytes exacerbate tissue injury through excessive cytokine production, release of reactive oxygen species, and formation of neutrophil extracellular traps, which further damage endothelial integrity and disrupt microcirculatory flow. Clinical studies have demonstrated associations between elevated circulating inflammatory mediators, endothelial injury markers, and adverse neurological and systemic outcomes following cardiac arrest.

**Figure 2:**
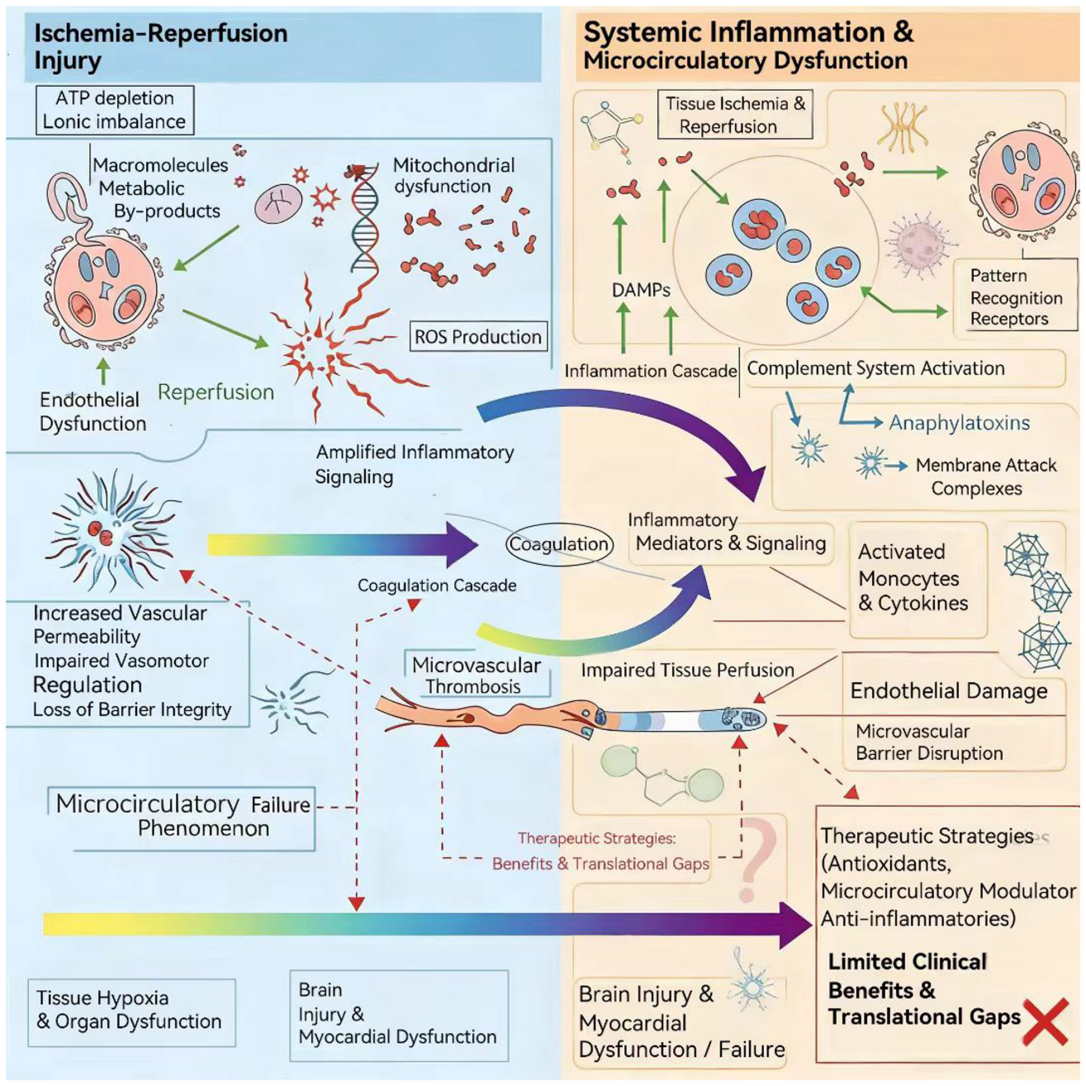
Schematic overview of the pathophysiological mechanisms of post-cardiac arrest syndrome (PCAS).

Despite strong experimental and observational evidence supporting a central role for systemic inflammation in PCAS, clinical trials targeting individual inflammatory mediators have yielded largely inconclusive results. These findings suggest that the inflammatory response in PCAS is highly redundant and multifactorial, limiting the effectiveness of single-target anti-inflammatory strategies and highlighting the need for integrative, systems-level therapeutic approaches (7, 9–12). The integrated pathophysiological interactions underlying post-cardiac arrest syndrome are schematically summarized in Figure 2.

## 3 Cellular and molecular mechanisms of post-cardiac arrest syndrome (PCAS)

### 3.1 Oxidative stress and free radical damage

Oxidative stress represents a central pathological feature of post-cardiac arrest syndrome (PCAS) and is primarily driven by excessive reactive oxygen species (ROS) generation during reperfusion. Following restoration of spontaneous circulation, abrupt reoxygenation disrupts mitochondrial electron transport, leading to electron leakage and rapid ROS overproduction. This oxidative burst occurs in parallel with impaired endogenous antioxidant defenses, resulting in a profound redox imbalance.

Excessive ROS generation during reperfusion induces widespread cellular injury, simultaneously damaging macromolecules, impairing mitochondrial function, and amplifying inflammatory signaling cascades. Mitochondrial dysfunction is both a source and a consequence of oxidative stress, as damaged mitochondria further exacerbate ROS production, reduce ATP synthesis, and promote release of pro-apoptotic factors. This self-amplifying cycle contributes to cellular energy failure and increases vulnerability to subsequent inflammatory and cell death pathways.

Beyond direct cytotoxic effects, oxidative stress plays a critical role in endothelial dysfunction and microcirculatory impairment following cardiac arrest. ROS-mediated injury to endothelial cells disrupts barrier integrity, alters vasoregulatory signaling, and promotes leukocyte adhesion, collectively impairing tissue perfusion despite restoration of macroscopic blood flow. These effects provide a mechanistic link between oxidative stress and multiple organ dysfunction in PCAS.

Despite consistent experimental evidence supporting oxidative stress as a key driver of PCAS-related injury, clinical trials targeting ROS through antioxidant therapies have yielded largely inconclusive results. These findings suggest that oxidative damage in PCAS is highly complex, temporally dynamic, and closely intertwined with inflammation and mitochondrial failure, thereby limiting the efficacy of isolated antioxidant strategies and highlighting the need for integrated, system-level therapeutic approaches (13–15).

### 3.2 Activation of cell death pathways

Post-cardiac arrest syndrome (PCAS) is characterized by extensive cellular injury involving the activation of multiple regulated cell death pathways. Rather than operating as isolated mechanisms, apoptosis, necroptosis, and pyroptosis are increasingly recognized as overlapping and interconnected responses to severe ischemia–reperfusion stress, oxidative damage, and inflammatory signaling.

Apoptosis is prominently observed in vulnerable tissues such as the brain and myocardium following cardiac arrest, primarily mediated through mitochondrial dysfunction, cytochrome c release, and caspase activation. In parallel, necroptosis, a caspase-independent form of regulated necrosis, contributes to membrane rupture and secondary inflammation, particularly under conditions where apoptotic pathways are impaired. Pyroptosis further amplifies tissue injury by promoting inflammasome activation, gasdermin-mediated membrane pore formation, and release of pro-inflammatory cytokines.

Importantly, these cell death pathways exhibit substantial molecular crosstalk and redundancy. Experimental studies demonstrate that inhibition of a single pathway often results in compensatory activation of alternative death programs, limiting the effectiveness of highly specific interventions. Endoplasmic reticulum stress and disrupted calcium homeostasis further integrate with mitochondrial and inflammatory signaling, serving as common upstream stressors that converge on multiple death pathways.

From a translational perspective, the simultaneous engagement of diverse and partially redundant cell death mechanisms helps explain why targeted modulation of individual pathways has not consistently translated into clinical benefit. These observations suggest that effective cytoprotective strategies in PCAS may require coordinated modulation of upstream stress responses—such as oxidative stress, mitochondrial dysfunction, and systemic inflammation—rather than selective inhibition of downstream execution pathways (16, 17).

## 4 PCAS animal model research

### 4.1 Common animal model construction methods

Post-cardiac arrest syndrome (PCAS) animal models are primarily established using two main methods: ventricular fibrillation (VF) induction and asphyxia induction. The ventricular fibrillation induction method involves electrically triggering VF to simulate sudden cardiac arrest, which closely mimics the sudden onset of arrhythmias seen in clinical cardiac arrest cases. This approach allows for precise control of the timing and severity of cardiac arrest and has been widely used to study the pathophysiology of PCAS, including post-cardiac arrest brain injury (PCABI), myocardial dysfunction, and systemic ischemia/reperfusion injury (IRI) (6). On the other hand, the asphyxia method induces cardiac arrest by causing hypoxia, often through airway obstruction or respiratory arrest, representing a different clinical scenario such as drowning or respiratory failure. Both methods are valuable for exploring different aspects of PCAS pathogenesis and therapeutic interventions.

Large animal models, particularly pigs, offer significant advantages in hemodynamic studies of PCAS due to their physiological and anatomical similarities to humans. Porcine models allow for detailed cardiovascular monitoring, including invasive hemodynamic measurements and imaging, providing insights into systemic and cerebral perfusion changes during and after cardiac arrest. Moreover, their size permits the use of clinical devices and resuscitation protocols, enhancing translational relevance (6). Conversely, small animal models, such as rodents, are extensively utilized for molecular mechanism studies due to their genetic manipulability, cost-effectiveness, and availability of molecular tools. Rodent models facilitate investigations into cellular and molecular pathways involved in PCAS, including oxidative stress, inflammation, and neuronal apoptosis. Although small animals lack the hemodynamic fidelity of large animals, their use complements large animal studies by enabling mechanistic explorations at the molecular level (6).

In summary, the construction of PCAS animal models utilizes ventricular fibrillation and asphyxia induction methods, each reflecting distinct clinical cardiac arrest scenarios. Large animal models, especially pigs, are preferred for hemodynamic and resuscitation studies, while small animal models are indispensable for elucidating molecular mechanisms underlying PCAS. The integration of these models provides a comprehensive platform for understanding PCAS pathophysiology and developing effective therapies.

### 4.2 Model evaluation criteria and limitations

Evaluating PCAS animal models involves assessing key indicators such as the rate of return of spontaneous circulation (ROSC) and neurological function scores. The ROSC rate is a critical parameter reflecting the success of resuscitation efforts and the model's ability to simulate clinical cardiac arrest recovery. Neurological function scoring, often based on established scales adapted for animal use, provides a measure of brain injury severity and functional outcome post-resuscitation. These assessments are essential for validating the model's relevance to human PCAS and for evaluating therapeutic interventions (6).

However, different animal models exhibit variability in simulating human PCAS features. For instance, ventricular fibrillation-induced models may better replicate sudden cardiac arrest but may not encompass the hypoxic injury component seen in asphyxia-induced models. Additionally, species differences affect the pathophysiological responses; large animals like pigs have cardiovascular and neurological systems more analogous to humans, whereas small rodents may show divergent responses due to anatomical and physiological disparities. Such differences impact the translatability of findings and necessitate careful selection of models based on research objectives (6).

Standardization of PCAS models is crucial to enhance the comparability and reproducibility of research outcomes. Variations in induction methods, resuscitation protocols, and evaluation criteria across studies hinder the consolidation of data and the development of universally accepted therapeutic strategies. Establishing standardized protocols, including consistent definitions of ROSC, neurological assessment tools, and timing of evaluations, would improve the reliability of PCAS research. Moreover, addressing limitations such as the inability to fully replicate the complexity of human PCAS and the ethical considerations of animal use remains imperative for advancing the field (6).

In conclusion, PCAS animal models are evaluated based on ROSC rates and neurological function, yet differences among models in replicating human PCAS pose challenges. Standardization of modeling and evaluation protocols is essential to improve research comparability and translational potential. Recognizing and addressing these limitations will facilitate the development of effective interventions for PCAS.

## 5 Biomarker research in post-cardiac arrest syndrome (PCAS)

### 5.1 Neurological injury-related biomarkers

#### 5.1.1 Clinical application of neuron-specific enolase (NSE) and S100B Protein

Neuron-specific enolase (NSE) and S100B protein are well-established biomarkers reflecting neuronal injury and glial activation, respectively, and have been extensively studied in the context of neurological damage following cardiac arrest. NSE, a glycolytic enzyme found predominantly in neurons, is released into cerebrospinal fluid (CSF) and blood upon neuronal cell damage. Similarly, S100B, a calcium-binding protein mainly expressed by astrocytes, serves as a marker of glial injury and blood-brain barrier (BBB) disruption. Elevated serum or CSF levels of NSE and S100B have been correlated with the severity of brain injury and poor neurological outcomes in patients after cardiac arrest. For example, studies have demonstrated that higher NSE concentrations in serum and CSF are associated with worse functional outcomes and increased mortality in this population. However, the clinical utility of NSE is limited by methodological issues, including variability in assay specificity and interference from hemolysis, which can affect measurement accuracy. It has been suggested that current enzyme-linked immunosorbent assay (ELISA) methods may not adequately differentiate NSE isoforms or assess enzymatic activity, leading to conflicting results in clinical studies. S100B protein levels have also been shown to increase after cardiac arrest, reflecting astrocytic damage and BBB compromise. The temporal dynamics of these biomarkers are critical; for instance, elevated levels at 24 to 72 h post-insult are more predictive of outcomes. Despite their promise, NSE and S100B are best used in combination with clinical assessment and neuroimaging to improve prognostic accuracy. Advances in biosensor technologies, such as electrochemical immunosensors utilizing nanomaterials, have enhanced the sensitivity and rapidity of NSE detection, potentially facilitating point-of-care applications. Overall, NSE and S100B remain valuable neuroinjury biomarkers in PCAS, but standardization of assays and integration with multimodal monitoring are necessary to optimize their clinical application (18–21).

#### 5.1.2 Advances in neurofilament protein biomarkers

Neurofilament proteins, particularly neurofilament light chain (NfL) and phosphorylated neurofilament heavy chain (pNfH), have emerged as sensitive biomarkers of axonal injury and neurodegeneration. In PCAS, elevated levels of NfL in CSF and blood correlate with the extent of neuronal damage and have been shown to predict neurological outcomes and mortality. The high sensitivity of novel assays, such as single-molecule array (Simoa), allows detection of low concentrations of neurofilaments in peripheral blood, facilitating less invasive monitoring. Studies indicate that NfL levels rise following cardiac arrest and remain elevated during the acute and subacute phases, reflecting ongoing neuroaxonal injury. Moreover, pNfH has demonstrated prognostic value in various neurological conditions, although its role in PCAS is less defined. The combination of neurofilament measurements with other biomarkers like NSE and S100B may enhance prognostic precision. Importantly, neurofilament levels are influenced by blood-brain barrier integrity and systemic factors, necessitating careful interpretation. The development of mass spectrometry-based methods has further refined neurofilament quantification, providing accurate and reproducible assessments across neurodegenerative diseases. Incorporating neurofilament biomarkers into PCAS management could improve early detection of brain injury severity and guide therapeutic decisions (22–25).

#### 5.1.3 Cerebrospinal fluid biomarkers and blood-brain barrier integrity

The integrity of the blood-brain barrier (BBB) plays a pivotal role in the pathophysiology of PCAS and influences the release and clearance of neurological biomarkers. Disruption of the BBB facilitates the passage of brain-derived proteins such as NSE, S100B, and neurofilaments into the systemic circulation. Biomarkers like soluble platelet-derived growth factor receptor beta (sPDGFRβ) and syndecan-1 have been investigated as indicators of BBB damage in neurological injuries. Elevated levels of these markers in CSF and blood post-cardiac arrest correlate with worse neurological outcomes and may reflect endothelial glycocalyx degradation. Additionally, inflammatory cytokines and chemokines contribute to BBB dysfunction and secondary brain injury. Recent studies have emphasized the importance of assessing BBB integrity alongside neuroinjury biomarkers to better understand the extent of cerebral damage and predict recovery potential. The dynamic changes in BBB-associated biomarkers during the acute and subacute phases of PCAS provide insights into ongoing neurovascular injury and repair mechanisms (26–29)44r.

### 5.2 Systemic injury-related biomarkers

#### 5.2.1 High-sensitivity cardiac troponin in myocardial injury assessment

High-sensitivity cardiac troponin (hs-cTn) assays have revolutionized the detection of myocardial injury by enabling quantification of troponin at very low concentrations with high precision. In the context of PCAS, myocardial injury is common due to ischemia-reperfusion injury and systemic inflammatory responses. Elevated hs-cTn levels post-cardiac arrest are associated with adverse cardiac events and increased mortality. The kinetics of hs-cTn release, including peak levels and temporal changes, provide important prognostic information. However, interpretation of hs-cTn elevations must consider confounding factors such as chronic cardiac conditions and renal impairment. The use of sex-specific 99th percentile upper reference limits improves diagnostic accuracy, and serial measurements enhance risk stratification. Moreover, combining hs-cTn with other biomarkers, such as inflammatory markers or natriuretic peptides, may further refine prognostication. Clinical trials have demonstrated that hs-cTn-guided management can optimize therapeutic interventions in acute cardiac injury settings. Nonetheless, standardized protocols for hs-cTn use in PCAS remain to be established (30–33).

#### 5.2.2 Inflammatory cytokine profiles and endothelial injury markers

Systemic inflammation is a hallmark of PCAS, contributing to multiorgan dysfunction and poor outcomes. Pro-inflammatory cytokines such as interleukin-6 (IL-6), tumor necrosis factor-alpha (TNF-α), and interleukin-1 beta (IL-1β) exhibit dynamic changes following cardiac arrest, reflecting the severity of the inflammatory response. Elevated levels of these cytokines correlate with neurological injury and systemic complications. Additionally, endothelial injury markers, including syndecan-1 and soluble thrombomodulin, indicate vascular endothelial damage and glycocalyx shedding, which exacerbate capillary leak and organ dysfunction. The systemic immune-inflammation index (SII) and systemic inflammation response index (SIRI), which integrate counts of neutrophils, lymphocytes, and monocytes, have been proposed as composite markers reflecting the balance of pro- and anti-inflammatory states. These indices show promise in predicting adverse outcomes post-cardiac arrest and may guide immunomodulatory therapies. However, the timing of measurement and the interplay with BBB integrity and neuroinflammation require further elucidation (26, 34, 35).

#### 5.2.3 Emerging biomarkers and multimodal approaches

Recent advances in proteomics and metabolomics have identified novel biomarkers associated with systemic injury in PCAS, including markers of oxidative stress, endothelial dysfunction, and metabolic derangements. For instance, elevated levels of osteopontin, YKL-40, and neurofilament proteins in blood and CSF have been linked to systemic inflammatory states and organ injury. The integration of these biomarkers with clinical scoring systems and neuroimaging enhances the precision of prognosis. Furthermore, machine learning algorithms incorporating dynamic biomarker changes improve risk stratification and individualized patient management. The development of point-of-care assays for rapid biomarker detection holds promise for timely therapeutic decisions. Nonetheless, large-scale prospective studies are needed to validate these emerging biomarkers and to establish standardized protocols for their clinical use in PCAS (36–38).

## 6 Potential therapeutic targets of post-cardiac arrest syndrome (PCAS)

It should be emphasized that, with the exception of guideline-supported supportive strategies, the majority of therapeutic approaches discussed in this section remain investigational. Most available evidence is derived from experimental models or early-phase clinical studies, while robust randomized controlled trial data in patients with post-cardiac arrest syndrome are currently limited. Accordingly, the following discussion aims to clearly distinguish between preclinical mechanistic promise and established clinical evidence.

### 6.1 Interventions targeting ischemia-reperfusion injury

Ischemia-reperfusion injury (IRI) is a pivotal pathological process underlying the systemic and organ-specific damage observed in post-cardiac arrest syndrome (PCAS). The abrupt restoration of blood flow following cardiac arrest triggers a cascade of oxidative stress, inflammation, and cellular dysfunction, exacerbating tissue injury beyond the initial ischemic insult. Experimental preclinical studies have extensively investigated free radical scavengers and antioxidants as therapeutic agents to mitigate oxidative damage during IRI. For instance, the administration of antioxidants such as edaravone, a free radical scavenger, has shown promise in reducing oxidative stress and neuronal injury in cerebral ischemia-reperfusion models (39). Similarly, remimazolam, an ultra-short-acting benzodiazepine, demonstrates direct scavenging activity against hydroxyl radicals and superoxide anions, comparable to edaravone, suggesting potential utility in attenuating free radical-mediated damage in IRI (40). Beyond conventional antioxidants, novel synthetic compounds like phosphorochalcogenoates exhibit potent antioxidant and free radical scavenging activities with favorable safety profiles, representing a new class of agents for oxidative injury (41).

Mitochondria-targeted therapeutic strategies have emerged as a cutting-edge approach to address the central role of mitochondrial dysfunction in IRI. Mitochondria are both sources and targets of reactive oxygen species (ROS) during reperfusion, and their preservation is critical for cellular survival. Recent preclinical studies highlight the importance of maintaining mitochondrial homeostasis, with interventions aimed at modulating mitochondrial dynamics, biogenesis, and mitophagy. For example, the SENP1/SIRT3 axis has been identified as a key regulator in intestinal IRI, where reducing SENP1 expression alleviates mitochondrial damage and oxidative stress (42). Similarly, the nuclear receptor coactivator 4 (NCOA4)-mediated ferritinophagy pathway has been implicated in cerebral IRI, with iron chelators like deferoxamine attenuating injury by inhibiting this pathway (43). Pharmacological agents such as normobaric hyperoxia have been shown to activate the Keap1-Nrf2 pathway, enhancing antioxidant defenses and reducing renal IRI (44). Moreover, electroacupuncture interventions modulate gut microbiota and may attenuate systemic inflammation, thereby providing cardioprotection against myocardial IRI (45).

Microcirculatory protection is another critical therapeutic target in IRI, as microvascular dysfunction contributes to tissue hypoxia and organ failure. Agents that improve endothelial function, inhibit leukocyte adhesion, and preserve capillary integrity have demonstrated efficacy in experimental models. For instance, low-intensity pulsed ultrasound targeted at the spleen modulates splenic neuro-immune pathways, reducing endothelial dysfunction and inflammation in autoimmune myocarditis, which shares pathophysiological features with IRI (46). Additionally, small-molecule agonists of formyl peptide receptors (FPRs), such as compound 17b, exhibit vasodilatory and anti-inflammatory effects in pulmonary vasculature, highlighting the potential of targeting microcirculation in IRI-related conditions (47). Collectively, these experimental advances underscore the multifaceted nature of IRI and the necessity of integrated therapeutic strategies encompassing free radical scavenging, mitochondrial preservation, and microvascular protection to ameliorate PCAS.

### 6.2 Anti-Inflammatory and Immunomodulatory Strategies

Inflammation is a central component of PCAS pathophysiology, driven by innate immune activation, cytokine release, and complement system engagement following ischemia-reperfusion. Targeting inflammatory pathways offers a promising avenue to mitigate secondary injury and improve outcomes. The complement system, particularly components such as C1q, C3a, and C5a, plays a dual role in mediating inflammation and tissue repair. Inhibition of complement activation has been shown to reduce neuroinflammation and improve neurological outcomes in brain ischemia models (48). Recombinant C1 esterase inhibitor (C1INH) exhibits neuroprotective effects by attenuating complement-mediated injury in cerebral ischemia-reperfusion. Moreover, complement modulation has therapeutic potential in hepatic IRI, where targeted inhibitors of C3 and C5 pathways ameliorate liver damage while preserving regenerative capacity (49).

Cytokines are pivotal mediators of the inflammatory response in PCAS. Interleukin-1 (IL-1) family cytokines, including IL-1β and IL-1 receptor antagonist (IL-1ra), are rapidly upregulated following ischemic injury and contribute to neuroinflammation and cell death. Experimental evidence supports the use of IL-1 receptor antagonists such as anakinra to reduce inflammation and improve neurological outcomes after traumatic brain injury and stroke (50, 51). The modulation of IL-1 signaling represents a targeted approach to dampen excessive inflammation without compromising host defense. Additionally, other pro-inflammatory cytokines such as IL-6, TNF-α, and IL-18 are implicated in PCAS and may serve as biomarkers or therapeutic targets (52, 53).

Macrophages and microglia are key cellular effectors in PCAS-associated inflammation. Their polarization states (M1 pro-inflammatory vs. M2 anti-inflammatory) influence tissue injury and repair. Strategies that promote macrophage polarization toward the M2 phenotype have demonstrated efficacy in reducing inflammation and enhancing tissue regeneration in various ischemic models (54, 55). Nanomedicine approaches enabling targeted delivery of immunomodulatory agents to macrophages are under investigation to fine-tune the inflammatory milieu (56). Furthermore, emerging evidence suggests that modulation of immune checkpoints and signaling pathways such as Notch and NF-κB can influence immune cell function and inflammation in PCAS (57, 58).

Regulatory T cells (Tregs) also hold therapeutic potential due to their immunosuppressive functions. Enhancing Treg activity may attenuate detrimental inflammation and promote tissue healing in PCAS, as suggested by preclinical studies in other inflammatory conditions (52). Complementary approaches including dietary interventions, traditional Chinese medicine, and electroceutical modalities have shown promise in modulating systemic and local inflammation in ischemia-reperfusion contexts (59, 60). Overall, anti-inflammatory and immunomodulatory strategies targeting cytokines, complement, and immune cell phenotypes represent a multifaceted therapeutic paradigm to ameliorate PCAS and improve patient outcomes.

Taken together, current therapeutic strategies targeting ischemia–reperfusion injury, oxidative stress, and inflammation in post-cardiac arrest syndrome are largely supported by experimental and early-phase clinical evidence. With the exception of guideline-supported interventions such as targeted temperature management, most approaches remain investigational, underscoring the substantial translational gap between mechanistic insights and clinically validated therapies. A comparative overview of major therapeutic strategies, their mechanistic rationale, supporting evidence, and translational status is provided in Table 1.

**Table 1:** Comparison of major therapeutic strategies in post-cardiac arrest syndrome.

**Therapeutic strategy**	**Mechanistic rationale**	**Preclinical evidence**	**Clinical trial evidence**	**Current translational status**
Free radical scavengers	Attenuation of reperfusion-associated oxidative stress	Consistent reduction of cellular and organ injury in animal cardiac arrest models	Randomized trials have not demonstrated consistent improvements in neurological outcome or survival	Investigational
Mitochondrial-targeted therapies	Preservation of mitochondrial function and ATP production; reduction of mitochondrial ROS	Protective effects observed in multiple experimental models	Limited to early-phase or exploratory clinical studies	Investigational
Anti-inflammatory strategies	Modulation of cytokine signaling and inflammatory cascades	Reduced tissue injury and organ dysfunction in experimental studies	Clinical trials targeting single inflammatory mediators show largely inconclusive results	Investigational
Immunomodulatory approaches	Restoration of immune homeostasis after systemic ischemia–reperfusion	Beneficial immunophenotypic shifts in preclinical models	Insufficient clinical evidence	Experimental
Targeted temperature management	Reduction of metabolic demand and secondary brain injury	Neuroprotection consistently demonstrated in experimental models	Supported by randomized clinical trials and international guidelines	Standard of care

## 7 PCAS brain protection research

### 7.1 Mechanisms of brain ischemic tolerance

Brain ischemic tolerance refers to the phenomenon where brief, non-lethal ischemic episodes induce resistance to subsequent severe ischemic insults, providing neuroprotection. This endogenous protective mechanism involves complex molecular and cellular processes, including ischemic preconditioning, postconditioning, and pharmacological preconditioning. Ischemic preconditioning (IPC) has been extensively studied in cerebral ischemia models, demonstrating that it can activate multiple signaling pathways to reduce neuronal apoptosis and inflammation, maintain blood-brain barrier (BBB) integrity, and promote tissue repair. For example, electroacupuncture (EA) pretreatment induces cerebral ischemic tolerance by downregulating pro-inflammatory factors such as HES1 and NF-κB, thereby attenuating cortical neuronal injury after ischemia-reperfusion (61). EA also mediates neuroprotection by activating astroglial cannabinoid type 1 receptors (CB1R), leading to moderate glutamate upregulation that protects neurons from ischemic injury (62). Moreover, hypoxic preconditioning (HPC) can alleviate receptor interacting protein 3 (RIP3)-induced necroptosis in hippocampal CA1 neurons, thereby regulating microglia/macrophage polarization and reducing neuroinflammation (63). These findings highlight the importance of modulating neuronal death pathways and glial responses in ischemic tolerance.

At the molecular level, ischemic tolerance involves epigenetic regulation and signaling cascades. For instance, the downregulation of Piwil2 by HPC prevents cerebral ischemic injury by inhibiting CREB2 promoter methylation, leading to improved dendritic complexity and cognitive function (64). The unfolded protein response (UPR), particularly the activating transcription factor 6 (ATF6) pathway, contributes to cerebral ischemia-reperfusion injury by modulating endoplasmic reticulum stress and oxidative damage, suggesting potential therapeutic targets (65). Additionally, the matrix metalloproteinase (MMP) family, especially MMP-9, plays a dual role in BBB disruption and repair during ischemic events, with modulation of MMP activity being crucial for maintaining barrier integrity (66). The balance between pro-apoptotic and anti-apoptotic signaling, including the regulation of decoy receptors that inhibit TRAIL-mediated apoptosis, also underlies IPC-induced neuroprotection (67).

Furthermore, endogenous molecules such as 25-hydroxycholesterol (25-HC) can protect against cerebral ischemia-reperfusion injury by inhibiting stimulator of interferon gene (STING) activity, thereby reducing autophagy and neuronal apoptosis (68). Natural compounds like curcumin and protocatechuic acid (PCA) have demonstrated antioxidative, anti-inflammatory, and anti-apoptotic effects that contribute to neuroprotection in ischemic models (69, 70). These agents modulate signaling pathways including HIF-1α/VEGFA and cAMP-PKA-CREB, enhancing neuronal survival and functional recovery.

In summary, brain ischemic tolerance is a multifaceted process involving modulation of apoptotic pathways, inflammatory responses, epigenetic regulation, and BBB preservation. Therapeutic strategies that mimic or enhance these endogenous protective mechanisms, including preconditioning paradigms and pharmacological agents, hold promise for improving outcomes after cerebral ischemia.

### 7.2 Blood-brain barrier injury and repair

The blood-brain barrier (BBB) is a critical interface that maintains central nervous system homeostasis by regulating molecular and cellular trafficking between the blood and brain parenchyma. BBB disruption is a hallmark of post-cardiac arrest syndrome (PCAS) and cerebral ischemia, contributing to neuroinflammation, edema, and neuronal injury. Matrix metalloproteinases (MMPs), particularly MMP-9, are key mediators of BBB breakdown through degradation of tight junction proteins and extracellular matrix components (66, 71). The regulation of MMP activity, including their induction by extracellular matrix metalloproteinase inducer (EMMPRIN/CD147), is complex and temporally dynamic during brain injury (71, 72). Inhibition of MMPs has been shown to reduce BBB permeability and improve neurological outcomes in preclinical models (73).

Tight junction proteins such as claudins, occludin, and zonula occludens-1 (ZO-1) are essential for BBB integrity. Their expression and localization are dynamically regulated during injury and repair. For example, electroacupuncture treatment can preserve BBB integrity by modulating histone acetylation at MMP-9 and TIMP-2 gene promoters, thereby balancing proteolytic activity and tight junction protein expression (74). Natural compounds like trilobatin and resveratrol also protect BBB function by inhibiting MMP-9 expression and inflammatory signaling pathways (75, 76). Moreover, vasoactive intestinal peptide (VIP) upregulation mitigates anesthesia- and surgery-induced BBB disruption and cognitive impairment in aged rats by modulating inflammation and barrier integrity (77).

Emerging evidence highlights the role of mitochondrial oxidative stress in brain microvascular endothelial cells as a trigger for BBB disruption, suggesting that targeting mitochondrial dysfunction may be a viable therapeutic strategy (78). Additionally, the interaction between microglia and endothelial cells influences BBB permeability; microglial activation can exacerbate barrier breakdown via inflammasome activation and chemokine production, promoting leukocyte infiltration (79). Conversely, fibroblasts expressing Col1α1 contribute to BBB repair after intracerebral hemorrhage through TIMP2-dependent mechanisms (80).

Advanced age and sex differences also affect BBB resilience to oxidative stress and injury, with implications for PCAS and stroke outcomes (81, 82). Furthermore, the gut-brain axis and systemic factors such as hyperhomocysteinemia can modulate BBB permeability via matrix metalloproteinase activation and tight junction disruption (83, 84).

At the molecular level, post-translational modifications of tight junction proteins, including phosphorylation and ubiquitination, regulate their assembly and barrier function, offering potential targets for therapeutic intervention (85). Structural studies of claudin proteins reveal that subtle sequence variations influence polymerization and tight junction dynamics, which are critical for barrier maintenance (86).

In therapeutic contexts, stem cell-derived extracellular vesicles (EVs) have shown promise in repairing BBB damage by delivering bioactive molecules that modulate inflammation, oxidative stress, and neurovascular regeneration (87, 88). Nanotechnology-based approaches, including nanoparticle-mediated drug delivery and exosome engineering, offer innovative strategies to overcome BBB limitations and enhance treatment efficacy for hemorrhagic stroke and other CNS disorders (89, 90).

Overall, BBB disruption in PCAS and cerebral ischemia involves a complex interplay of proteolytic enzymes, tight junction remodeling, inflammatory responses, and cellular interactions. Advances in understanding these mechanisms provide a foundation for developing targeted therapies to preserve and restore BBB integrity, ultimately improving neurological outcomes.

## 8 PCAS research: new technologies and methods

### 8.1 Application of omics technologies

Omics technologies, encompassing genomics, transcriptomics, proteomics, and metabolomics, have revolutionized biomedical research by enabling comprehensive, high-throughput profiling of biological molecules. In the context of post-cardiac arrest syndrome (PCAS), these approaches offer unprecedented opportunities to unravel the complex molecular underpinnings driving the pathophysiology and to identify novel diagnostic and prognostic biomarkers. Transcriptomics, through RNA sequencing and gene expression profiling, reveals differential gene expression patterns characteristic of PCAS, highlighting pathways involved in inflammation, apoptosis, and metabolic dysregulation (91). Proteomics complements this by identifying alterations in protein abundance and post-translational modifications, uncovering novel biomarkers that may correlate with disease severity or therapeutic response (92). Metabolomics, by profiling small-molecule metabolites, reflects the dynamic changes in energy metabolism and oxidative stress that are hallmarks of PCAS (93). Integration of these omics layers through multi-omics approaches further enhances the ability to construct comprehensive molecular networks, elucidating interactions among genes, proteins, and metabolites that drive PCAS progression (94). Single-cell and spatial omics technologies add spatial and cellular resolution, enabling the dissection of heterogeneity within affected tissues, such as the brain, and revealing cell-type-specific responses to ischemia-reperfusion injury (95). Moreover, advances in bioinformatics and machine learning facilitate the analysis and integration of multi-omics data, improving biomarker discovery and enabling predictive modeling of patient outcomes (96). Despite these advances, challenges remain, including standardization of sample processing, data analysis, and validation of findings in clinical cohorts. Nonetheless, the application of omics technologies holds great promise for advancing the understanding of PCAS pathogenesis and for developing precision medicine strategies tailored to individual patient profiles.

### 8.2 Advances in imaging technologies

Imaging technologies have undergone significant advancements, providing critical insights into the structural and functional consequences of PCAS, particularly in the brain. Functional magnetic resonance imaging (fMRI), including resting-state and task-based paradigms, enables noninvasive assessment of cerebral blood flow, oxygenation, and neural activity alterations following cardiac arrest (97). Functional MRI techniques such as blood oxygen level-dependent (BOLD) imaging allow evaluation of brain injury severity and recovery by mapping changes in neuronal activation and connectivity patterns (98). Positron emission tomography (PET) imaging with tracers like 18F-fluorodeoxyglucose (FDG) provides metabolic imaging of cerebral glucose utilization, revealing hypometabolic regions associated with neuronal injury and prognosticating neurological outcomes (99). Recent developments in PET tracers targeting synaptic density, neuroinflammation, and mitochondrial function further enhance the specificity of molecular imaging in PCAS (100). Near-infrared spectroscopy (NIRS), including broadband NIRS and diffuse correlation spectroscopy (DCS), offers portable, bedside monitoring of cerebral oxygenation, blood flow, and mitochondrial metabolism, facilitating continuous assessment in critical care settings (101). Integration of multimodal imaging, combining PET, MRI, and NIRS, provides complementary information on cerebral perfusion, metabolism, and structural integrity, improving diagnostic accuracy and monitoring of therapeutic interventions (101). Additionally, emerging imaging techniques such as magnetic resonance elastography (MRE) and advanced optical imaging methods expand the capabilities for detecting subtle tissue changes post-PCAS (102). The application of artificial intelligence and machine learning to imaging data analysis further enhances the sensitivity and specificity of detecting brain injury and predicting outcomes (96). Collectively, these imaging advances enable comprehensive evaluation of PCAS-related brain injury, guiding clinical decision-making and informing the development of targeted therapies.

## 9 Conclusion

Post-cardiac arrest syndrome (PCAS) represents a multifaceted systemic pathophysiological condition characterized by the intricate interplay among multiple organs and systems. From an expert perspective, understanding the development and impact of PCAS requires a comprehensive integration of diverse research findings that elucidate its complex mechanisms and therapeutic challenges.

Central to the pathogenesis of PCAS are ischemia-reperfusion injury, inflammatory responses, and oxidative stress. These core mechanisms not only drive the initial tissue damage following cardiac arrest but also perpetuate secondary injury processes that complicate patient recovery. The dynamic and interconnected nature of these mechanisms underscores the necessity of adopting a holistic approach in both research and clinical management. Balancing the contributions of each pathological factor is critical, as interventions targeting one pathway may inadvertently influence others, emphasizing the need for precision in therapeutic design.

Animal models have been indispensable in advancing our understanding of PCAS, providing controlled environments to dissect molecular and cellular pathways involved in post-arrest injury. However, the current heterogeneity in animal model protocols limits the reproducibility and translational potential of findings. Standardization of these models is imperative to ensure that preclinical results are robust and applicable to human conditions. This harmonization will facilitate more accurate evaluation of novel interventions and enhance the predictive value of animal studies.

The advent of multi-omics technologies and advanced imaging modalities has revolutionized PCAS research by enabling comprehensive profiling of molecular alterations and real-time visualization of organ function. These cutting-edge tools allow for the identification of novel biomarkers and therapeutic targets, offering unprecedented insights into the temporal and spatial dynamics of PCAS. Integrating multi-omics data with clinical parameters holds promise for developing personalized medicine approaches that tailor interventions to individual patient profiles, thereby optimizing outcomes.

Targeted therapeutic strategies addressing specific pathological components of PCAS are emerging as promising avenues to improve patient prognosis. Interventions aimed at mitigating ischemia-reperfusion injury, modulating inflammatory cascades, and attenuating oxidative stress have shown potential in preclinical studies. Nonetheless, translating these strategies into effective clinical treatments remains challenging due to the complexity of PCAS and patient heterogeneity. A balanced consideration of the interplay among various therapeutic targets is essential to avoid adverse effects and maximize clinical benefits.

Looking forward, future research must prioritize translational medicine to bridge the gap between basic science discoveries and clinical application. This entails rigorous validation of preclinical findings in human studies, development of standardized protocols, and implementation of multidisciplinary collaborations. Emphasizing translational efforts will accelerate the incorporation of innovative diagnostic and therapeutic modalities into routine clinical practice, ultimately enhancing survival and neurological outcomes for patients suffering from PCAS.

In conclusion, the evolution of PCAS research reflects a growing appreciation of its systemic complexity and the necessity for integrated investigative approaches. By harmonizing diverse research perspectives—from mechanistic studies and animal models to multi-omics analyses and targeted therapies—experts can forge a path toward more effective management strategies. Continued commitment to translational research will be pivotal in transforming scientific insights into tangible clinical advancements, thereby improving the prognosis and quality of life for patients affected by this challenging syndrome.
